# Determination of a cut-off COmprehensive Score for financial Toxicity (COST) for identifying cost-related treatment nonadherence and impaired health-related quality of life among Chinese patients with cancer

**DOI:** 10.1007/s00520-024-08320-w

**Published:** 2024-01-27

**Authors:** Binbin Xu, Winnie K. W. So, Kai Chow Choi

**Affiliations:** 1 School of Nursing, Hunan University of Chinese Medicine, Changsha, China; 2 The Nethersole School of Nursing, The Chinese University of Hong Kong, Hong Kong, China

**Keywords:** COmprehensive Score for financial Toxicity, Cut-off score, Cost-related treatment nonadherence, Health-related quality of life, Cancer, Chinese

## Abstract

**Purpose:**

This study aimed to determine a cut-off for the simplified Chinese version of the COmprehensive Score for financial Toxicity (COST) that could identify cost-related treatment nonadherence among Chinese patients with cancer. The study also sought to validate this cut-off score by using it to assess impaired health-related quality of life (HRQoL) in the same population.

**Methods:**

A secondary analysis was conducted using data from a cross-sectional survey of 1208 Chinese patients with cancer who were recruited from 12 hospitals in six cities across three provinces of the Chinese mainland. Sociodemographic information and data on financial toxicity (FT), cost-related treatment nonadherence, and HRQoL were used in the analysis. Receiver operating characteristic (ROC) analysis was used to determine the optimal cut-off for the simplified Chinese version of the COST.

**Results:**

The ROC analysis identified a COST cut-off of 18.5 for identifying cost-related treatment nonadherence, yielding a sensitivity of 76.5% and specificity of 71.4%. In the validation study, this cut-off score yielded a sensitivity of 64.2% and a specificity of 67.1% for identifying impaired HRQoL.

**Conclusion:**

Early and dynamic assessment of cancer-related FT in routine clinical practice may play a crucial role in the early identification and management of FT. Accordingly, a COST cut-off of 18.5 was identified to indicate cost-related treatment nonadherence and impaired HRQoL in a population of patients with cancer from the Chinese mainland. This finding may facilitate the implementation of universal FT screening among patients with cancer in specific settings such as the Chinese mainland.

## Introduction

Patients with cancer bear significant economic burdens due to direct medical and non-medical costs and indirect costs. Substantial direct medical costs are often incurred because of the multifaceted nature of cancer treatment, which may include surgery, chemotherapy, radiation therapy, immunotherapy, and targeted therapy [1, 2]. Diagnostic tests and supportive care further contribute to high direct medical costs, particularly for patients who lack adequate health insurance coverage [3–6]. Increasing survival rates have led to longer treatment durations, which have further increased patients’ direct medical costs [7]. Even after completing treatment, patients continue to face high direct medical costs related to follow-up care, ongoing medications, and surveillance testing. Direct non-medical costs, such as specialised dietary supplements, transportation, accommodation, and informal nursing, also can quickly accumulate and thus pose significant challenges for patients with limited financial resources [3, 5, 6]. Additionally, indirect costs related to the impact of the illness on patients’ ability to earn income and caregivers’ lost productivity further compound the economic burden associated with cancer [3, 4, 6]. Extended periods of unemployment can have a severe financial impact on patients and their families [8].

American scholars coined the term ‘financial toxicity’ (FT) to describe the economic side effects of cancer care [9]. Studies have shown that FT is prevalent among patients with cancer in both developed and developing countries. In the USA, a systematic review showed that 39–64% of adult patients with cancer had experienced FT [10]. Another study found that 7–48% of patients with cancer in countries with publicly funded healthcare systems had experienced FT, although their out-of-pocket (OOP) expenses were lower than those of patients in the USA [11]. In the Chinese mainland, the prevalence of FT among adult patients with cancer was reported to range from 6 to 84% [12].

An emerging body of evidence consistently highlights the impact of FT on patients’ ability to access medical care and their health-related quality of life (HRQoL). For example, patients may compromise their adherence to treatments (e.g., refuse to take prescribed drugs or stop attending clinics) to save money [13]. A meta-analysis revealed that patients with cancer who had higher levels of FT were nearly twice the likelihood of medication nonadherence and more than twice the odds of poor overall physical, mental, emotional, and social functioning well-being than patients with lower levels of FT [10].

In recognition of these detrimental impacts, universal screening has been suggested as a potential strategy to reduce the risk of FT among patients with cancer [14]. By identifying patients at risk of FT at an early stage, healthcare providers can develop customised management plans that may include educational interventions [15], financial navigation programmes [16], financial counselling programmes [17], multi-disciplinary stepped psychosocial care programmes [18], personalised health insurance decision aids [19], and cost conversation aids [20]. The integration of screening FT into standard care for patients with cancer is recommended.

The COmprehensive Score for financial Toxicity (COST), a patient-reported outcome measure, is the most commonly used validated instrument to measure FT in patients with cancer [13, 21]. COST has been translated into more than 10 languages. The simplified Chinese version can be accessed online (https://www.facit.org/measures/FACIT-COST) and has exhibited good validity and reliability, with a Cronbach’s α of 0.89, test–retest reliability of 0.77–0.98, a scale content validity index of 0.82, and item content validity index between 0.83 and 1.00 [22, 23]. However, a cut-off for the simplified Chinese version of COST has not yet been identified, limiting the clinical applicability of this score for FT screening among Chinese patients with cancer.

Therefore, the current study aimed to identify a cut-off score for the simplified Chinese version of COST that would facilitate screening for cost-related treatment nonadherence among patients with cancer in the Chinese mainland. Additionally, the study sought to validate the identified cut-off score in assessing impaired HRQoL in the same patient population. The information from this study provides valuable insights to inform the clinical screening of FT among patients with cancer and to facilitate early assessments and interventions. By establishing a COST cut-off score, healthcare providers can identify patients at risk of FT and initiate target interventions to mitigate its adverse effects, potentially improving treatment adherence and HRQoL among Chinese patients with cancer.

## Materials and methods

### Study design

This study was a secondary analysis of data collected during the first author’s PhD thesis project, which involved a cross-sectional study and primarily aimed to investigate the current FT status of Chinese patients with cancer and the associated factors. The study used a multistage stratified sampling method to recruit participants from 12 hospitals in six cities across three provinces of the Chinese mainland. First, the three provinces were randomly selected such that each represented a high-, middle-, or low-income region in the Chinese mainland; then, two cities were randomly chosen in each selected province to represent high- and low-income cities. Next, one tertiary and one secondary hospital were randomly selected from each of the six chosen cities, yielding a total of 12 hospitals. In the Chinese mainland, hospitals are classified into three tiers, namely primary, secondary, and tertiary, based on their size and the level of services provided [24]. Primary hospitals are typically township or community hospitals with fewer than 100 beds; these hospitals provide preventive care, minimal health care, and rehabilitation services to a single community. Secondary hospitals are usually affiliated with medium-sized cities, counties, or districts and have between 100 and 500 beds; they provide comprehensive health services to multiple communities. Tertiary hospitals are comprehensive, referral, and general hospitals at the city, provincial, or national level that have bed capacities exceeding 500. These hospitals provide specialist health services and serve as medical hubs, providing care to multiple regions.

The dataset for this analysis was obtained from an existing PhD thesis project. The original data were collected through face-to-face e-surveys using validated questionnaires, including COST to assess FT and other measures to evaluate cost-related treatment nonadherence, HRQoL, as well as associated factors. Ethical approval for the original collection of data was obtained from the Survey and Behavioural Research Ethics Committee of the Chinese University of Hong Kong (Reference No. SBRE-21–0403). All procedures performed in this study were in accordance with the Declaration of Helsinki. All of the participants provided informed consent before being included in the study.

From February to October 2022, 1208 participants were recruited from the 12 selected hospitals and completed the survey. The participants were adult patients who had been diagnosed with cancer at any site and stage and who had received active anticancer treatment for at least two consecutive months [25] or had completed initial treatment. Approximately one-third of the participants were each from high-income (*n* = 408, 33.8%), middle-income (*n* = 400, 33.1%), and low-income (*n* = 400, 33.1%) regions. The ratio of participants from tertiary hospitals (*n* = 1008) to those from secondary hospitals (*n* = 200) was approximately 5:1. The data of all 1208 participants were included in the secondary analysis.

### Variables and measures used in the secondary analysis

#### Participants’ general characteristics

Participants provided detailed information on their sociodemographic factors, socioeconomic status, and disease and treatment characteristics. The sociodemographic information included the participant’s age at cancer diagnosis, sex, marital status, and residence; the socioeconomic information included the participant’s education level, annual household income, employment status before cancer diagnosis, and type of social health insurance; and the disease and treatment-related characteristics included the cancer site, cancer stage, duration since cancer diagnosis, and treatment plan.

#### FT

FT was measured using the simplified Chinese version of COST [26], which consists of 12 items to assess patients’ perception of financial distress. Item 12 is a summary item used for screening that was not scored. The other 11 items were scored using a 5-point Likert scale ranging from 0 (‘Not at all’) to 4 (‘Very much’). Items 1, 6, 7, and 11 were considered positive items and scored directly, whereas the other items used negative wording and were reverse-scored. The scores of each item were then summed to obtain the total score (possible range: 0–44), with a lower score indicating higher FT. The Chinese version of COST has exhibited good validity and reliability, with a Cronbach’s α of 0.89 and a scale content validity index of 0.82 [22, 23].

#### Cost-related treatment nonadherence

Cost-related treatment nonadherence was measured using a single question to ask whether a participant had ever delayed, foregone, or made other changes to their cancer care because of the cost. The following six types of care were assessed: prescription medicine, visits to specialists, treatment (other than prescription medicine), follow-up care, mental health services, and ‘other’. This question was taken from the Medical Expenditure Panel Survey (MEPS): Your Experiences with Cancer [27], and it has been used in previous studies [28, 29]. The MEPS: Your Experiences with Cancer is a self-administered questionnaire developed by scholars from the National Cancer Institute, Centers for Disease Control and Prevention, the American Cancer Society, Agency for Healthcare Research and Quality, and Westat [30, 31]. This questionnaire was developed based on a systematic review of existing survey instruments that assess cancer survivorship, and it underwent several rounds of rigorous cognitive testing among approximately 60 cancer survivors with different levels of educational attainment, types of health insurance, employment status, and time since cancer diagnosis [30]. A dichotomous summary measure of cost-related treatment nonadherence was generated when a patient responded with ‘Yes’ regarding any cancer care that they had delayed or foregone because of the cost [28, 29].

#### HRQoL

HRQoL was measured using the simplified Chinese version of the Functional Assessment of Cancer Therapy-General (FACT-G; Version 4), which contains 27 items in four domains: physical well-being, social/family well-being, emotional well-being, and functional well-being [32]. A 5-point Likert scale ranging from 0 (‘Not at all’) to 4 (‘Very much’) was used to score the items [32]. The subscale and total scores were computed by summing the item scores. The possible ranges of scores for the four subscales and the overall scale were 0–28, 0–28, 0–24, 0–28, and 0–108, respectively. Higher scores indicate better health [32]. The FACT-G total score was utilized for the analysis in the present study. The simplified Chinese version of FACT-G has good internal consistency, with a Cronbach’s α greater than 0.80 for all subscales [33].

### Statistical methods

Statistical analysis was conducted using IBM SPSS 26.0 (IBM Corp., Armonk, NY, USA). Descriptive statistics, including the frequency, percentage, mean, standard deviation (SD), median, and interquartile range (IQR), were used to summarise the participants’ general characteristics and their FT, cost-related treatment nonadherence, and HRQOL outcomes. The normality of continuous variables was assessed using skewness and kurtosis statistics, which yielded acceptable absolute values of ≤ 2 and ≤ 7, respectively [34]. Receiver operating characteristic (ROC) analysis [35] was conducted to examine the ability of the COST to identify cost-related treatment nonadherence. The area under the ROC curve (AUC) is a measure of the diagnostic ability of a test (i.e., the COST) to correctly classify cases with and without a specified outcome (i.e., cost-related treatment nonadherence). An AUC value of 1.0 indicates perfect diagnostic accuracy, and a value of ≤ 0.5 suggests an uninformative diagnosis [35]. AUC values of 0.7–0.8, 0.8–0.9, and > 0.9 are considered acceptable, excellent, and outstanding, respectively [36]. The COST value corresponding to the point on the ROC curve nearest to the upper left corner was identified as the optimal cut-off score for the identification of cost-related treatment nonadherence, as this point maximised both sensitivity and specificity with equal weighting and hence maximised the Youden index. The derived cut-off score was further validated in an assessment to identify impaired HRQoL, which conventionally has been defined as a FACT-G total score in the first quartile [25].

## Results

### Participant characteristics

Table 1 summarises the participants’ sociodemographic, socioeconomic, and disease and treatment-related characteristics. The participants had a mean age of 53.53 years (SD = 11.74) at cancer diagnosis, and nearly half were male (51.3%, *n* = 620). The majority of the participants were married (90.5%, *n* = 1093), and over half resided in rural areas (57.5%, *n* = 695). The majority of participants had a below-undergraduate education level (92.0%, n = 1111). Before their cancer diagnosis, the participants were engaged in various occupations, including self-employment or farming (36.2%, *n* = 437), retirement or unemployment (32.0%, *n* = 386), and employment (31.9%, *n* = 385). More than 60% of the participants had an annual household income below 80,000 Chinese yuan (62.2%, *n* = 751). Almost all of the participants had social medical insurance (99.4%, *n* = 1201). The most common cancer diagnoses were lung cancer (22.0%, *n* = 266) and colorectal cancer (14.4%, *n* = 174). Approximately half of the participants had advanced cancer (50.7%, *n* = 613), and the majority had received combination therapy (88.9%, *n* = 1074). The median time from cancer diagnosis to the survey was 7 months (IQR: 4.00–19.00 months).

**Table 1:** General characteristics of the participants (*N* = 1208)

Characteristics	*n* (%)
Age at cancer diagnosis [Mean (Standard deviation)]	53.53 (11.74)
Sex	
Female	588 (48.7)
Male	620 (51.3)
Marital status	
Married	1093 (90.5)
Single/Divorced/Widowed	115 (9.5)
Residence	
Rural area	695 (57.5)
Urban area	513 (42.5)
Educational level	
Primary school or below	373 (30.9)
Junior high school	362 (30.0)
High school or technical secondary school	255 (21.1)
Junior college	121 (10.0)
Undergraduate or above	97 (8.0)
Employment status before cancer diagnosis	
Self-employed/farmer	437 (36.2)
Employed: Civil servants/Employees of public institutions or state-owned enterprises	99 (8.2)
Employed: Employees of private enterprises/workers	286 (23.7)
Retired/unemployed	386 (32.0)
Annual household income (Chinese Yuan)	
< = 30,000	335 (27.7)
> 30,000 and < = 60,000	230 (19.0)
> 60,000 and < = 80,000	186 (15.4)
> 80,000 and < = 150,000	253 (20.9)
> 150,000	204 (16.9)
Social medical insurance	
No social medical insurance	7 (0.6)
Urban and rural resident basic medical insurance	692 (57.3)
Urban employee basic medical insurance	509 (42.1)
Specific sites of the cancer	
Lung	266 (22.0)
Colorectal	174 (14.4)
Head and neck	168 (13.9)
Breast	149 (12.3)
Gastrointestinal (excluding colorectal)	148 (12.3)
Gynaecological	130 (10.8)
Haematological	94 (7.8)
Others ^a^	79 (6.5)
Cancer stage	
I	62 (5.1)
II	149 (12.3)
III	353 (29.2)
IV	613 (50.7)
Unknown	31 (2.6)
Received combination therapy	
No	134 (11.1)
Yes	1074 (88.9)
Duration since cancer diagnosis (months) [Median (Inter-quartile range)]	7.00 (4.00–19.00)

^a^ Other cancer types included brain/central nervous system cancer (*n* = 20), sarcoma (*n* = 15), prostate cancer (*n* = 12), bladder cancer (*n* = 10), kidney cancer (*n* = 6), skin cancer (*n* = 6), testicular cancer (*n* = 3), mesothelioma (*n* = 2), bone cancer (*n* = 2), ureteral cancer (*n* = 1), thymoma (*n* = 1), and melanoma of mucosal (*n* = 1)

### COST cut-off for identifying cost-related treatment nonadherence and impaired HRQoL

In this study, the mean COST score for FT was 20.53 (SD = 6.70), and the mean FACT-G score for HRQoL was 59.26 (SD = 12.86). Based on our criteria, 25.7% of participants were categorised as having impaired HRQoL, with a FACT-G total score falling within the first quartile (determined to be 51 in our study) serving as the threshold for impairment. Additionally, 25.7% of the participants reported treatment nonadherence because of cancer care costs.

**Fig. 1:**
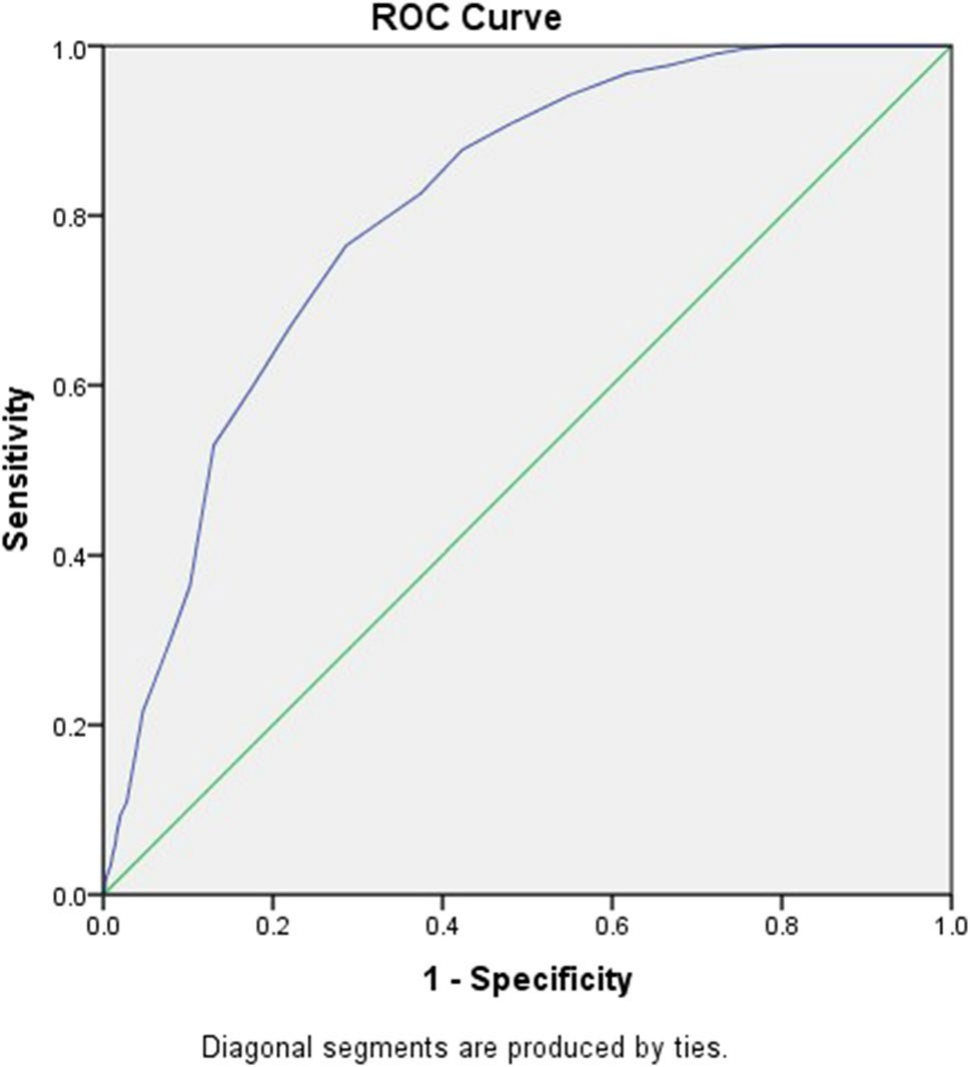
Receiver operating characteristic curve; sensitivity is plotted against one minus specificity, with cost-related treatment nonadherence as the external criterion and an area under the curve of 0.806 (*p* < 0.001)

**Fig. 2:**
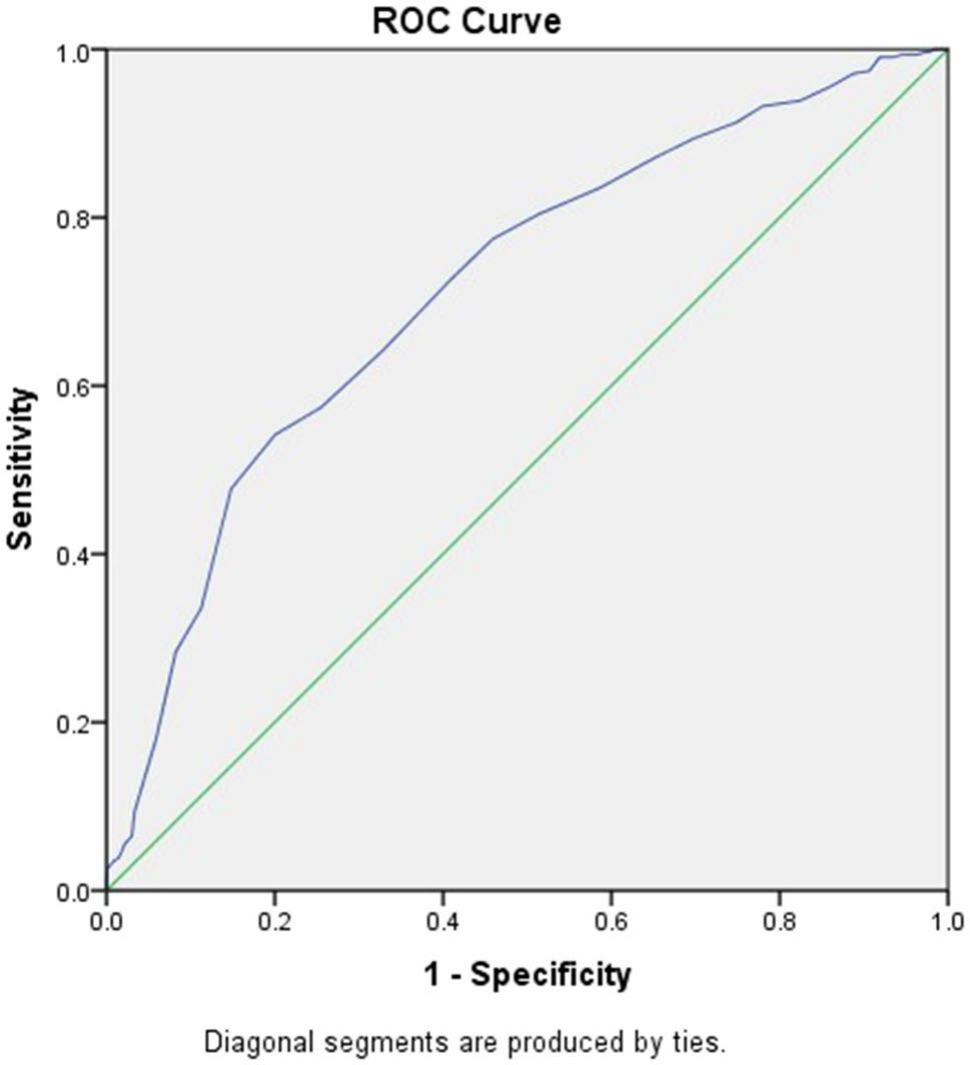
Receiver operating characteristic curve; sensitivity is plotted against one minus specificity, with low health-related quality of life as the external criterion and an area under the curve of 0.716 (*p* < 0.001)

Figure 1 presents the ROC curve generated using cost-related treatment nonadherence as the external criterion. The AUC value was 0.806 (*p* < 0.001), indicating excellent diagnostic performance. The ROC analysis results (Table 2) suggested that a cut-off COST of 18.5 could offer a good balance between sensitivity (76.5%) and specificity (71.4%) and a maximum Youden index of 47.8%, thereby enabling the identification of treatment nonadherence resulting from cancer care costs. Furthermore, the cut-off score of 18.5 yielded a sensitivity of 64.2% and a specificity of 67.1% for identifying impaired HRQoL. The AUC of the COST cut-off for diagnosing impaired HRQoL was 0.72 (*p* < 0.001; Fig. 2), indicating acceptable diagnostic performance.

**Table 2:** Coordinates of the receiver operating characteristic curve with cost-related treatment nonadherence as the external criterion

Cutoff scores ^a^	Sensitivity (%)	Specificity (%)	Youden Index (%)
0.0	0.0	100.0	0.0
1.5	0.0	99.9	− 0.1
2.5	0.3	99.9	0.2
3.5	0.6	99.8	0.4
4.5	1.3	99.8	1.1
5.5	1.9	99.7	1.6
6.5	3.5	99.1	2.7
7.5	4.8	98.9	3.7
8.5	6.1	98.6	4.7
9.5	7.1	98.4	5.5
10.5	9.4	98.0	7.4
11.5	11.0	97.2	8.2
12.5	21.6	95.3	16.9
13.5	29.7	92.2	21.9
14.5	36.5	89.8	26.2
15.5	52.9	87.0	39.9
16.5	60.3	82.1	42.4
17.5	67.1	77.8	44.9
18.5	76.5	71.4	47.8
19.5	82.6	62.6	45.2
20.5	87.7	57.7	45.4
21.5	90.6	52.3	43.0
22.5	94.2	45.0	39.2
23.5	96.8	38.2	35.0
24.5	97.7	33.2	30.9
25.5	99.0	27.8	26.9
26.5	99.7	24.3	24.0
27.5	100.0	19.7	19.7
28.5	100.0	15.7	15.7
29.5	100.0	12.2	12.2
30.5	100.0	10.2	10.2
31.5	100.0	8.5	8.5
32.5	100.0	7.0	7.0
33.5	100.0	5.7	5.7
34.5	100.0	5.0	5.0
35.5	100.0	3.9	3.9
36.5	100.0	2.8	2.8
37.5	100.0	2.3	2.3
38.5	100.0	1.6	1.6
39.5	100.0	1.2	1.2
40.5	100.0	0.9	0.9
41.5	100.0	0.3	0.3
42.5	100.0	0.2	0.2
43.5	100.0	0.1	0.1
45.0	100.0	0.0	0.0

^a^ The smallest cutoff value is the minimum observed test value minus 1, and the largest is the maximum value plus 1. All the other cutoff values are the averages of two consecutive ordered observed test values

## Discussion

This study is the first to establish a cut-off for COST in the context of the Chinese mainland. Specifically, we identified a cut-off score of 18.5 for the simplified Chinese version of COST. The effectiveness of this cut-off score for detecting treatment nonadherence resulting from cancer care costs among Chinese patients with cancer was demonstrated by a sensitivity of 76.5% and specificity of 71.4%. Furthermore, the established cut-off score also satisfactorily identified impaired HRQoL in the same population.

Previous COST-based studies on FT in the Chinese mainland utilised a cut-off score of 26 [37–39] or 17.5 [40]. These cut-off scores were determined using data from USA [41] and Hong Kong [25] populations, respectively. Additionally, several studies have used the median COST [42] or half of the total COST (i.e., 22) [43] as a cut-off. However, whether these cut-off scores can identify cost-related treatment nonadherence and HRQoL impairment among Chinese patients with cancer requires consideration. To the best of our knowledge, no evidence exists to support the use of half of the total COST (i.e., 22) as a cut-off score when assessing Chinese patients with cancer. Additionally, the use of the median COST appears to lack scientific rigour, as differences in the median score across studies would make it difficult to compare the results of multiple studies.

Furthermore, a cut-off score of 26 or 17.5 may not be applicable in the Chinese mainland because of differences between healthcare systems in the USA, Hong Kong, and the Chinese mainland. Specifically, in the Chinese mainland, healthcare is provided by both the public and private sectors, with public medical institutions serving as the primary providers. Healthcare costs are funded by multiple financial sources, including central and local government health expenditures, capital investment in health services by all sectors of society (e.g., medical insurance paid by enterprises for employees, commercial insurance companies), and individuals’ OOP expenses [44]. Individual OOP health expenditures accounted for 27.6% of the total health expenditures in the Chinese mainland in 2021 [44] despite the availability of universal health coverage. Actual hospitalisation reimbursement rates for the urban employee basic medical insurance and urban and rural resident basic medical insurance programmes were reported to be 76% and 60%, respectively, in 2019 [45]. The USA has a hybrid healthcare system with multiple revenue sources [46], similar to that used in the Chinese mainland. However, most healthcare facilities in the USA are privately owned and operated, and no universal healthcare coverage is available [46]. A report indicated that one in 10 Americans were uninsured [46]. Although Hong Kong is part of China, it has a distinct health care system because of differences in economic foundations, social systems, and ideologies [47]. As a Special Administrative Region of China, Hong Kong enjoys a high degree of autonomy that has enabled it to design and implement its own policies and systems, including healthcare. The healthcare system in Hong Kong was developed within the context of a market-oriented economic framework supported by robust social security and healthcare service systems [47]. The Hong Kong government was reported to offer nearly free healthcare to all citizens [47], and tax-based financing pays for 90% of medical costs [25].

The COST cut-off score determined in the current study could provide acceptable sensitivity and specificity for the screening of treatment nonadherence because of cancer care costs and impaired HRQoL among patients with cancer in the Chinese mainland. For example, it could be used by a multidisciplinary cancer care team to screen susceptible patients with cancer and thus allow the prompt initiation of supportive services to alleviate the negative impact of FT on treatment adherence and HRQoL among patients with cancer. Integrating FT screening into the standard care provided to patients with cancer has been regarded as a prerequisite for FT management that could contribute to early identification and intervention [14]. However, FT assessment is typically not part of a routine clinical evaluation in the Chinese mainland [12]. The proposed cut-off score determined in this study could facilitate the implementation of universal FT screening among patients with cancer in specific settings such as the Chinese mainland.

### Limitations

The present study has several limitations. First, although we used a multistage sampling method to enhance the sample’s representativeness, a convenience sampling method was used in the final stage to recruit participants from the randomly selected hospitals because it was not feasible to compile a sampling frame for recruiting eligible participants in each selected hospital. This may have resulted in selection bias. For example, the majority of participants were diagnosed with stage III/IV cancer and may have received more treatment and experienced more severe FT than patients with less advanced disease. Second, our study used patient-reported outcome data, which may have led to recall bias. Finally, the cost-related treatment nonadherence was measured using a single question from the MEPS: Experiences with Cancer Questionnaire [27]. Although its widespread application and rigorous cognitive testing during development underscore its reliability [28–30], it is pertinent to acknowledge the limited availability of specific psychometric validation studies in the literature for this questionnaire. Furthermore, our treatment nonadherence measure was only a previous behaviour, which may not represent future behaviour, a properly chosen timeframe for the measure assessment should be carefully considered with a prospective study design. When interpreting the study’s findings or generalising them to other contexts, these limitations should be considered.

## Conclusion

Early and dynamic assessment of cancer-related FT in routine clinical practice may play a crucial role in the early identification and management of FT. Accordingly, in this study a COST cut-off of 18.5 was found to indicate treatment nonadherence due to cancer care costs and impaired HRQoL in the context of the Chinese mainland. These findings may facilitate the implementation of universal FT screening among patients with cancer in specific settings such as the Chinese mainland.

## Data Availability

The dataset generated during and/or analysed during the current study are available from the corresponding author on reasonable request.
